# Biallelic Variants in *PYROXD2* Cause a Severe Infantile Metabolic Disorder Affecting Mitochondrial Function

**DOI:** 10.3390/ijms23020986

**Published:** 2022-01-17

**Authors:** Nicole J. Van Bergen, Daniella H. Hock, Lucy Spencer, Sean Massey, Tegan Stait, Zornitza Stark, Sebastian Lunke, Ain Roesley, Heidi Peters, Joy Yaplito Lee, Anna Le Fevre, Oliver Heath, Cristina Mignone, Joseph Yuan-Mou Yang, Monique M. Ryan, Colleen D’Arcy, Margot Nash, Sile Smith, Nikeisha J. Caruana, David R. Thorburn, David A. Stroud, Susan M. White, John Christodoulou, Natasha J. Brown

**Affiliations:** 1 Brain and Mitochondrial Research Group, Murdoch Children’s Research Institute, Royal Children’s Hospital, Parkville, VIC 3052, Australia; lucy.spencer@vcgs.org.au (L.S.); sean.massey@mcri.edu.au (S.M.); tegan.stait@mcri.edu.au (T.S.); david.thorburn@mcri.edu.au (D.R.T.); david.stroud@unimelb.edu.au (D.A.S.); 2 Department of Paediatrics, University of Melbourne, Parkville, VIC 3010, Australia; zornitza.stark@vcgs.org.au (Z.S.); sebastian.lunke@vcgs.org.au (S.L.); joy.lee@rch.org.au (J.Y.L.); Joseph.Yang4@rch.org.au (J.Y.-M.Y.); sue.white@vcgs.org.au (S.M.W.); 3 Department of Biochemistry and Pharmacology and Bio21 Molecular Science and Biotechnology Institute, The University of Melbourne, Parkville, VIC 3010, Australia; daniella.hock@unimelb.edu.au (D.H.H.); nikeisha.caruana@unimelb.edu.au (N.J.C.); 4 Victorian Clinical Genetics Services, Murdoch Children’s Research Institute, Parkville, VIC 3052, Australia; ain.roesley@vcgs.org.au (A.R.); anna.lefevre@vcgs.org.au (A.L.F.); 5 Australian Genomics Health Alliance, Parkville, VIC 3052, Australia; 6 Department of Pathology, University of Melbourne, Parkville, VIC 3010, Australia; 7 Department of Metabolic Medicine, Royal Children’s Hospital, Parkville, VIC 3052, Australia; heidi.peters@mcri.edu.au (H.P.); oliver.heath@rch.org.au (O.H.); 8 Medical Imaging Department, Royal Children’s Hospital, Parkville, VIC 3052, Australia; cristina.mignone@rch.org.au; 9 Department of Neurosurgery, Neuroscience Advanced Clinical Imaging Service (NACIS), The Royal Children’s Hospital, Parkville, VIC 3052, Australia; 10 Developmental Imaging, Murdoch Children’s Research Institute, Parkville, VIC 3052, Australia; 11 Neuroscience Research, Murdoch Children’s Research Institute, Parkville, VIC 3052, Australia; 12 Neurology Department, Royal Children’s Hospital, Parkville, VIC 3052, Australia; monique.ryan@rch.org.au; 13 Anatomical Pathology Department, Royal Children’s Hospital, Parkville, VIC 3052, Australia; colleen.darcy@rch.org.au; 14 General Medicine, Royal Children’s Hospital, Parkville, VIC 3052, Australia; margot.nash@rch.org.au; 15 Paediatric Intensive Care Unit, Royal Children’s Hospital, Parkville, VIC 3052, Australia; sile.smith@rch.org.au; 16 Institute for Health and Sport (iHeS), Victoria University, Footscray, VIC 3011, Australia; 17 Discipline of Child and Adolescent Health, University of Sydney, Camperdown, NSW 2006, Australia

**Keywords:** mitochondria, reactive oxygen species, genome sequencing, mitoribosome, oxidative phosphorylation, *PYROXD2*, ultrarapid genomics

## Abstract

Pyridine Nucleotide-Disulfide Oxidoreductase Domain 2 (*PYROXD2*; previously called *YueF*) is a mitochondrial inner membrane/matrix-residing protein and is reported to regulate mitochondrial function. The clinical importance of *PYROXD2* has been unclear, and little is known of the protein’s precise biological function. In the present paper, we report biallelic variants in *PYROXD2* identified by genome sequencing in a patient with suspected mitochondrial disease. The child presented with acute neurological deterioration, unresponsive episodes, and extreme metabolic acidosis, and received rapid genomic testing. He died shortly after. Magnetic resonance imaging (MRI) brain imaging showed changes resembling Leigh syndrome, one of the more common childhood mitochondrial neurological diseases. Functional studies in patient fibroblasts showed a heightened sensitivity to mitochondrial metabolic stress and increased mitochondrial superoxide levels. Quantitative proteomic analysis demonstrated decreased levels of subunits of the mitochondrial respiratory chain complex I, and both the small and large subunits of the mitochondrial ribosome, suggesting a mitoribosomal defect. Our findings support the critical role of *PYROXD2* in human cells, and suggest that the biallelic *PYROXD2* variants are associated with mitochondrial dysfunction, and can plausibly explain the child’s clinical presentation.

## 1. Introduction

Mitochondrial disease is a group of clinically heterogeneous conditions in which the optimal mitochondrial function is disrupted, causing a wide range of symptoms [1,2]. Mitochondrial disease can occur at any age, and manifest with a wide variety of symptoms that can affect many organs or tissues in an isolated fashion or systemically [3,4]. The heterogeneity of mitochondrial disease can be explained by pathogenic variants in either the mitochondrial genome (mtDNA) or nuclear DNA, and heterogeneity occurs even among family members with the same variant, leading to challenges in diagnosing and treating mitochondrial disease [1]. Mitochondrial dysfunction is associated with a wide range of conditions, including peripheral neuropathies, chronic kidney disease, aging related neurological disease, and cancers [5]. Typically, the most affected body systems are those with the highest energy demands, and thus the highest dependence on optimal mitochondrial function, such as the brain and muscles [3,4,6]. The common symptoms of mitochondrial disease in childhood include hypotonia, developmental delay, seizures, poor growth, and failure to thrive. The clinical biomarkers of mitochondrial disease include high levels of metabolic intermediates and enzymes or end products of anaerobic glucose metabolism due to the impairment of oxidative phosphorylation (OXPHOS), and can include lactate (lactic acidosis), pyruvate, and creatine kinase [3].

A challenge of diagnosing mitochondrial disease is the very broad clinical heterogeneity of disease presentation, which can delay diagnosis, and the number of disease-associated genes. However, the utilisation of next-generation sequencing (NGS) techniques, particularly the introduction of rapid genomic testing for critically ill paediatric patients, can substantially improve diagnostic rates and diagnostic time for critically ill infants [6,7,8] and greatly reduce the diagnostic odyssey of suspected mitochondrial disease patients [9]. Using NGS as a first line test for clinical diagnosis can immediately eliminate the need for several rounds of targeted sequencing, invasive testing (e.g., muscle biopsies), and lengthy functional studies. In some instances, such as new disease-associated genes, functional validation is often required to validate the genomic finding [1,3,10,11].

Here, we present the findings on biallelic variants in a novel disease gene, Pyridine Nucleotide-Disulfide Oxidoreductase Domain 2 (*PYROXD2;* previously called *YueF*), which were identified in a patient with suspected mitochondrial disease. The affected child presented with acute neurological deterioration and unresponsive episodes. The child previously experienced seizures and postnatal onset poor growth. Brain magnetic resonance imaging (MRI) revealed the bilateral symmetric foci of cytotoxic oedema within the dorsal midbrain, suggestive of either a toxic or metabolic cause. The clinical and imaging findings of the patient were thought to resemble one of the more common childhood mitochondrial diseases: Leigh syndrome (LS). Subsequent biochemical testing revealed extreme metabolic acidosis, further supporting mitochondrial impairment. Ultra-rapid trio genome sequencing (GS), including mtDNA analysis, was uninformative but identified the compound heterozygous variants in *PYROXD2* (NM_032709.2:c.1490dupC; p.(Val498Cysfs*79) and NM_032709.2:c.1276G > A; p.(Gly426Ser)). Quantitative proteomic analysis demonstrated the significant alterations to OXPHOS complex I subunit levels, and the decreased amounts of proteins belonging to the mitoribosome in the *PYROXD2* patient cells relative to controls, suggesting a mitoribosome defect. Functional analysis in patient fibroblasts revealed a heightened sensitivity to culturing in galactose media, a common indication of impaired mitochondrial activity and increased mitochondrial superoxide levels. In summary, we provide evidence that *PYROXD2* variants can contribute to mitochondrial impairment in patients and are the likely cause of the child’s clinical presentation.

## 2. Results

### 2.1. Case Description

The proband presented with fluctuating but progressive neurological deterioration on a background of postnatal-onset poor growth, microcephaly, central hypotonia, and a complex movement disorder. The child died at 6.5 months of age during his fifth prolonged hospital admission.

He was the second child to non-consanguineous Caucasian parents. His mother had a history of multiple first trimester miscarriages. He was born at 39 + 1 weeks gestation, after an uneventful prenatal course and required no resuscitation at birth. His birth parameters were in the normal range (weight 3.57 kg (67th centile), length 51 cm (72nd centile), and OFC 35.5 cm (80th centile)).

At 2 weeks of age, he was noted to have feeding difficulties and lost 15% of his birth weight. He had intermittent vomiting and increasingly unsettled behaviour. At 3–4 weeks of age, he presented with apparent life-threatening events (ALTE) consisting of acute hypotonia with colour change and mottled peripheries. Seizures were suspected based on abnormal eye movements and episodic stiffening. Levetiracetam was initiated but was ceased when, at 5 weeks of age, the electroencephalogram (EEG) and MRI brain were normal.

At 11 weeks of age, poor weight gain, central hypotonia, and significant head lag were apparent, while his peripheral tone, power, and deep tendon reflexes were normal. He had unusual tongue flickering movements. He had poor weight gain with a weight of 3.81 kg (−3.57 SD) and OFC of 37.5 cm (−1.89 SD).

Developmentally, he was able to fix and follow, smile socially, and turn to sound at 15 weeks. At this age, he was admitted to intensive care in extremis with circulatory shock and lactic acidosis.

**Figure 1:**
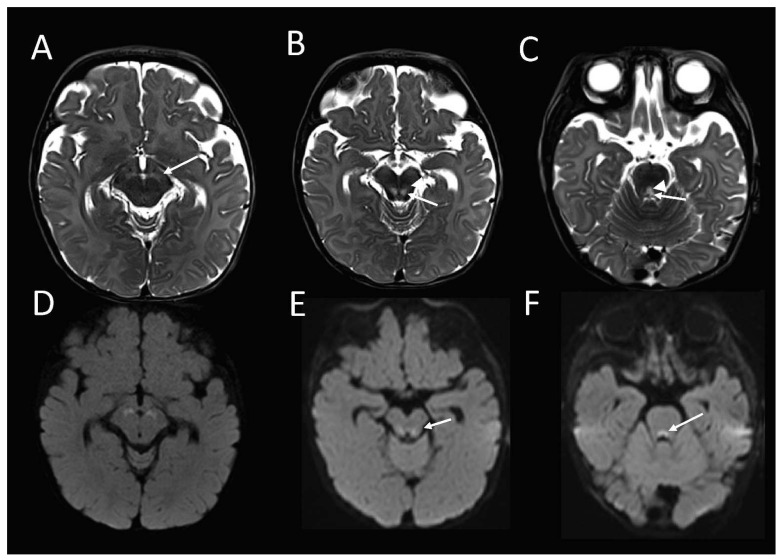
**Brain MRI from the patient with compound heterozygous *PYROXD2* variants at 4 ½ months**. The axial T2-weighted sequence shows abnormally high signal in the (**A**) substantia nigra; (**B**) midbrain tegmentum, involving the central tegmental tracts (arrow), and the medial lemnisci (arrowhead) and; (**C**) upper dorsal pontine tegmentum, involving the tectospinal tracts (arrowhead) and middle longitudinal fasciculi (arrow), with corresponding restricted diffusion (**D**–**F**) on diffusion-weighted imaging.

The brain MRI at 16 weeks of age demonstrated an abnormal high T2-weighted signal in the substantia nigra (Figure 1A), bilateral symmetric foci in the midbrain tegmentum, corresponding with the central tegmental tracts (CTT) and the medial lemnisci (ML) (Figure 1B), and the dorsal midline upper pons (Figure 1C), corresponding with the tectospinal tracts (TecSP) and the medial longitudinal fasciculus (MLF). These foci were associated with the restricted diffusion on diffusion-weighted imaging (Figure 1D–F), in keeping with the cytotoxic oedema. Spectroscopy that was performed at short follow-up 3 days later, was normal.

Following extubation, he developed repetitive dystonic movements and abnormal breathing. Reintubation was transiently required for bradycardia with associated apnoea. After discharge from intensive care at 18 weeks of age, dystonic posturing, and unusual mouth and tongue movements continued. Low dose gabapentin was commenced.

Evolving autonomic dysfunction led to abdominal distension and urinary voiding difficulties. From 20–25 weeks age, he developed increasing airway obstruction, decreased central tone, but increased peripheral muscle tone in association with brisk deep tendon reflexes. He had abnormal eye movements, described as upward flicking. A prolonged episode of airway obstruction at 25 weeks of age resulted in cardiac arrest, and he was readmitted to intensive care. At 27 weeks of age, there were features of a progressive movement disorder, including oromotor, upper and lower limb choreoathetosis, myoclonus, autonomic disturbance, and repeated airway obstruction.

**Figure 2:**
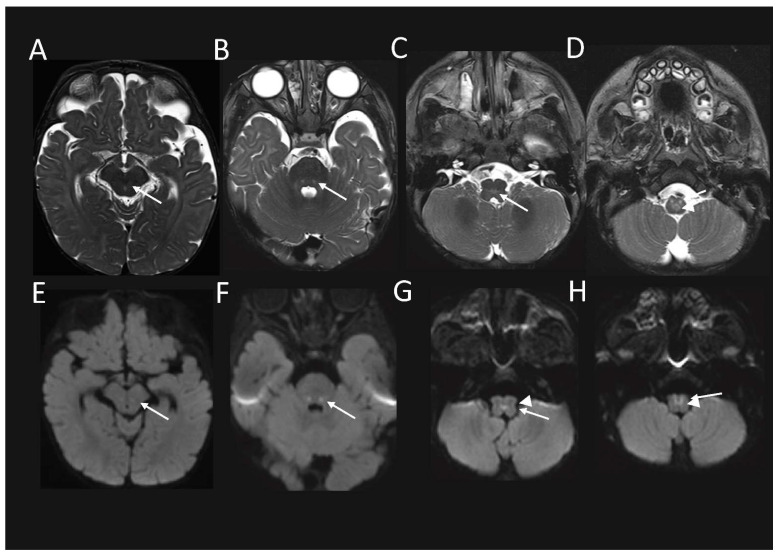
**Brain MRI from the patient with compound heterozygous *PYROXD2* variants at 6 months**. The axial T2-weighted sequence shows the interval development of abnormally high signal in the (**A**) red nuclei at the midbrain level; (**B**) central tegmental tracts and medial lemnisci (ML) (arrow), at the level of mid pons; (**C**) central dorsal medulla, involving the middle longitudinal fasciculi (MLF), and the tectospinal tracts (arrow), at the open medullary level; and (**D**) sensory decussation of ML (arrowhead), and MLF (arrow) at the upper part of closed medulla, with corresponding restricted diffusion (**E**–**H**) on diffusion-weighted imaging.

Follow up MRI performed just over 1.5 months later, demonstrated some interval signal improvement in the signal at the previously described sites. However, there were new abnormal high T2-weighted signal and associated restricted diffusion, and also developed in the red nuclei at the midbrain level (Figure 2A,E); in the CTT and ML at the mid pons level, (Figure 2B,F); in the MLF and TecSP (and possibly also ML) at the open medulla level (Figure 2C,G); and changes in the MLF and ML (i.e. the internal arcuate fibres of sensory decussation) at the upper part of the closed medulla, inferiorly (Figure 2D,H).

Extreme growth failure was evident with a weight of 3.88 kg (−6.38 SD), length of 65.5 cm (−1.21 SD), and OFC of 39 cm (−3.65 SD). Due to the clearly progressive and distressing nature of his condition, a decision was made to provide comfort care, and following a prolonged bradycardic episode, care was redirected towards palliation.

Genetic sequencing was provided via our Acute Care Genomics Flagship program [6], which provides rapid trio GS, including full mtDNA sequencing to critically ill children suspected of having a monogenic disorder. Blood samples were collected from the affected child and his parents for trio GS and RNAseq. A skin biopsy was performed for culture of fibroblasts for diagnostic respiratory chain enzymology, qPCR and RT-PCR RNA studies, and to study the functional impact of this variant on cell functioning.

Electron microscopy analysis of a glutaraldehyde-fixed, resin-embedded skeletal muscle biopsy collected from the affected child at the age of 6 months showed no definitive ultrastructural abnormalities. The muscle was comprised of myocytes with minimal variability in the myofibrillar size, and normal orientation. The Z-bands were unremarkable. Intracytoplasmic glycogen was present and of a normal quantity and distribution. The mitochondria in the biopsy were well-preserved and within the normal limits. There was no evidence of mitochondrial or lysosomal storage disease. Small intracellular lipid vacuoles were identified. No viral inclusions were identified and there were no tubuloreticular inclusions.

### 2.2. Genome Sequencing, RNAseq, and In Silico Analysis

The affected child and his unaffected parents underwent GS and RNA-seq analysis at the Victorian Clinical Genetics Services, Melbourne, Australia. No causative pathogenic variants were found through standard trio analysis of known disease-causing genes, including the analysis of mtDNA. The expanded analysis of research genes using Agilent Alissa Interpret, identified compound heterozygous variants in the gene encoding PYROXD2. The variants included a paternally inherited *PYROXD2* missense variant (NM_32709.2: c.1276G > A; p.(Gly426Ser); Supplemental Figure S1A), and a maternally inherited frameshift variant (NM_32709.2: c.1490dupC, p.(Val498Cysfs*79); Supplemental Figure S1B).

**Figure 3:**
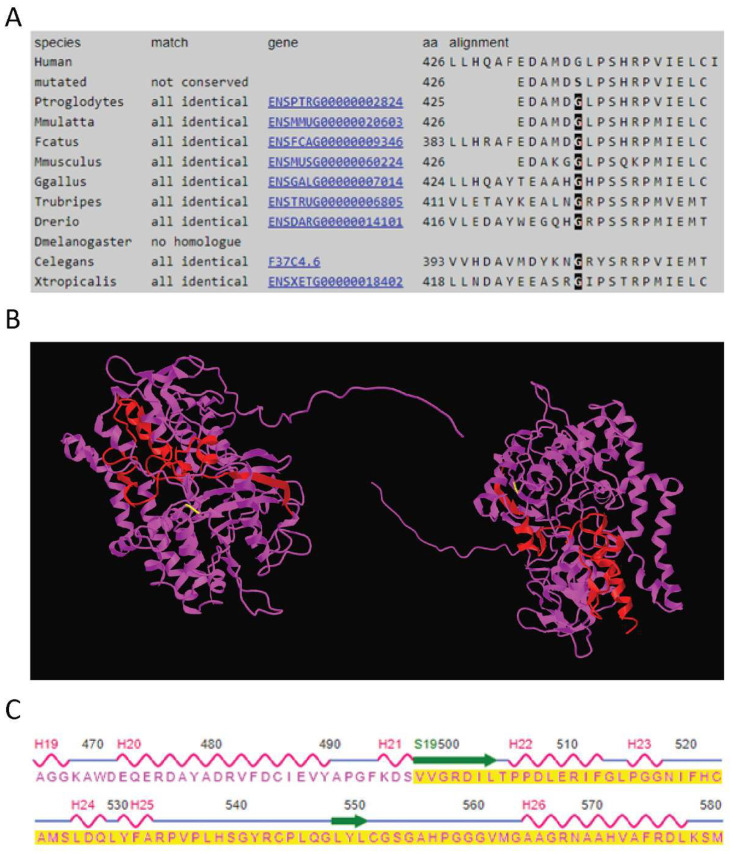
**Evolutionary sequence conservation and 3D structure of PYROXD2**. (**A**) The multiple sequence alignment of PYROXD2 reveals the affected p.Gly426 residue is highly conserved. (**B**) The predicted 3D structure of wild-type PYROXD2 (AF-Q8N2H3-F1, AlphaFold, modelled in icn3d) highlighting the patient variant p.(Gly426Ser) is highlighted in yellow, and the wild-type residues affected by the frameshift variant p.(Val498Cysfs*79) are highlighted in red. (**C**) Structural regions (from icn3d) indicate that the frameshift variant starting at p.Val498 (region highlighted in yellow) affects a beta-fold region (amino acids 498–504) and two major alpha-helical regions (amino acids 506–513 and 565–578) of the PYROXD2 protein.

The p.(Gly426Ser) missense variant is present in exon 12 of the *PYROXD2* gene and is predicted to be disease-causing by MutationTaster, and probably damaging (score of 0.994) and deleterious by PolyPhen-2 and SIFT analysis, respectively. This variant is present in gnomAD V2.1.1 with a global allele frequency of 8.41 × 10^−6^, with two heterozygous individuals of European (non-Finnish) ancestry and no homozygous individuals reported. The p.(Gly426Ser) variant lies within a highly conserved (Phast cons score of 0.997, Phylop score of 3.734) region of the protein, predicted to be an amino-oxidase domain of the protein (Figure 3A). The variant has not previously been published in the literature or relevant variant databases (ClinVar, HGMD public, DECIPHER, LOVD). RNAseq analysis confirmed the missense variant was expressed in the proband (Supplemental Figure S1C), in approximately 50% of the observed reads. The presence of the variant in the paternal RNA sample could not be confirmed due to the insufficient read depth.

The second variant introduces a change from valine to cysteine at codon 498 of the protein, followed by a frameshift that introduces a premature stop-codon 79 amino acids downstream. As the variant is located in the last exon of the *PYROXD2* gene (Exon 14), it is predicted to escape non-sense mediated decay (NMD). Although there are no reported domains in this region, this would represent a significant alteration to ~15% of the protein and may result in significant 3D structural alterations in the regions highlighted in the wild-type protein structure (Figure 3B). The regions affected by the frameshift variant include a beta strand and two alpha helices (Figure 3C). Consistent with NMD escape, the frameshift variant was observed in the RNASeq data (Supplemental Figure S1D) in both the proband and the mother, in approximately 50% of the reads. The overall *PYROXD2* expression levels were not significantly decreased compared to the controls. Furthermore, RT-PCR and qPCR showed normal levels of the *PYROXD2* transcript (Supplemental Figure S2), and Western blotting demonstrated normal levels of the PYROXD2 protein (Supplemental Figure S3). The variant was present in gnomAD V2.1.1 with an allele frequency of 3.98 × 10^−6^, with one heterozygote individual of European (non-Finnish) ancestry and no homozygote individuals reported. The variant has not previously been published in the literature or relevant variant databases (ClinVar, HGMD public, DECIPHER, LOVD).

### 2.3. Respiratory Chain Enzymes from PYROXD2 Patients

The analysis of the OXPHOS enzyme activities was performed, as previously described [10,11]. A skeletal muscle biopsy showed the activity of the enzymes within the reference range when expressed relative to citrate synthase (CS) and relative to complex II (CII). The respiratory chain enzymes were non-diagnostic and were not suggestive of a respiratory chain enzyme defect. However, respiratory chain enzyme activity in skeletal muscle homogenate was elevated when expressed relative to protein (Supplemental Table S1). Similarly, the activity of the enzymes in cultured patient fibroblasts was within the reference range when expressed relative to CS and CII; however, the activity was higher when normalized to protein (Supplemental Table S2). The results could thus be consistent with mitochondrial proliferation, but the significance is unclear.

To address whether the levels of representative protein subunits of the mitochondrial respiratory chain were altered, a premixed antibody cocktail for Western blotting was used to analyse the relative abundance of five OXPHOS complex subunits of the respiratory chain. While we noted slightly lower levels of cytochrome c oxidase subunit 2 (COXII) and NADH dehydrogenase [ubiquinone] 1 beta subcomplex subunit 8 (NDUFB8) in the proband relative to some controls, the variation in their abundance across samples led us to discount them, as well as to assume that the changes in the protein levels of other representative subunits of the respiratory chain complexes were insignificant (Supplemental Figure S4).

### 2.4. Quantitative Proteomics in Patient Fibroblasts

**Figure 4:**
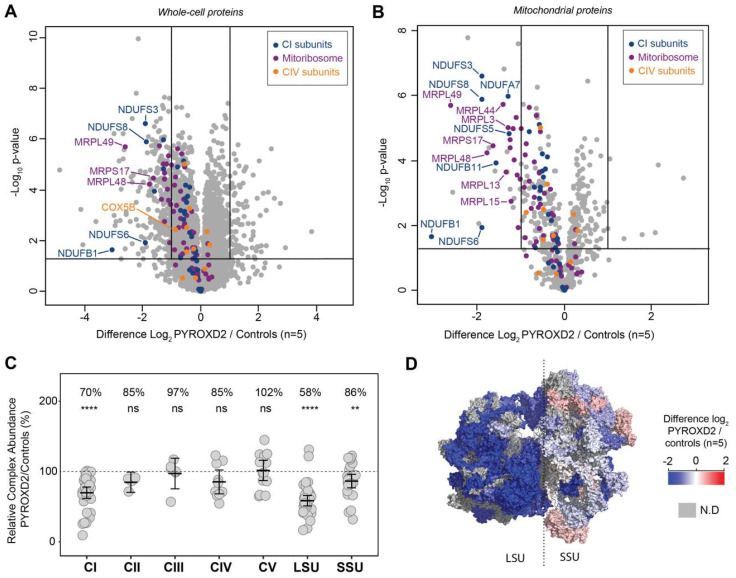
**Quantitative proteomics from patient fibroblasts indicate a complex I and mitoribosome defect**. Complex I subunits and proteins belonging to the mitoribosome are decreased in the *PYROXD2* patient relative to controls, suggesting a mitoribosome defect. (**A**) The volcano plot of the whole-cell fibroblast proteins, and the (**B**) volcano plot of the mitochondrial proteins (filtered in silico from MitoCarta 3.0 annotation) depicting the differences between the *PYROXD2* patient and the controls (*n* = 5). Significance lines were set to log2 = ±1 (equivalent to fold-change ±2) and *p*-value = 0.05 equivalent. (**C**) The relative complex abundance (RCA) plot of the OXPHOS complexes and mitoribosome subunits depicting CI, LSU, and SSU defects. The graph represents each complex ratio of PYROXD2/controls with a 95% confidence interval. *p*-value 0.05, **: *p* ≤ 0.01, ****: *p* ≤ 0.0001, ns: non-significant. (**D**) Topographical mapping of the relative abundances mapped to the mitoribosome structure depicting areas of decreased abundance. PDB id: 3J9M.

To obtain an overview of the proteome changes between the *PYROXD2* patient and the control fibroblasts, cell lysates from the patient and 5 off-pedigree control fibroblasts were subjected to tryptic digestion, followed by high-resolution tandem mass spectrometry (LC-MS/MS) and quantitative protein analysis. In total, we identified and quantified the abundance of 5068 proteins across all samples. Next, to determine the changes in the protein abundances, we compared the *PYROXD2* patient and control fibroblasts, and considered the proteins with a difference of log_2_ = ±1 (equivalent to fold-change ±2) and *p*-value 0.05 as being significant, represented in a volcano plot (Figure 4A). This analysis resulted in 263 proteins being considered of lower abundance in *PYROXD2* patient relative to control fibroblasts, and 87 proteins of higher abundance in *PYROXD2* patient compared to controls (Supplemental Table S3). PYROXD2 protein was not able to be quantified in the proteomic analysis, either in the patient or controls.

We then explored the dataset and performed gene ontology (GO) enrichment analysis [12,13,14] on the proteins that had significantly different levels (Supplemental Table S3). The 263 proteins of a lower abundance in the patient fibroblasts relative to the control fibroblasts, were analysed by GO enrichment analysis to determine the represented Reactome pathways. The top Reactome pathways of a lower abundance in the patient fibroblasts (Supplemental Table S4) were mitochondrial translation elongation (13.77-fold enrichment, FDR 9.71 × 10^−10^), mitochondrial translation termination (13.77-fold enrichment, FDR 4.85 × 10^−10^), mitochondrial translation initiation (13.77-fold enrichment, FDR 3.24 × 10^−10^), and complex-I biogenesis (11.01-fold enrichment, FDR 5.96 × 10^−4^). A volcano plot of the significantly altered proteins in the cell lysates (Figure 4A) highlighted both the mitoribosomal proteins involved in the mitochondrial translation (purple) and complex-I biogenesis (blue), which were significantly decreased in the patient cells. We then subset the data and only considered the mitochondrial proteins listed in the MitoCarta 3.0 database [15,16]. A volcano plot reveals the additional mitoribosomal and complex-I proteins as significantly decreased in the patient cells (Figure 4B).

The ratio of each OXPHOS complex subunit in the patient fibroblasts was normalized to that of the controls and showed a significant reduction in complex I (70% abundance) compared to the controls, whilst complexes II, III, IV, and V were normal (Figure 4C). A similar analysis for the mitoribosomal protein subunits of the small subunit (SSU) and large subunit (LSU) revealed a significant reduction in both the SSU and LSU. The topographical heatmapping of relative subunit abundances on to the cryo-electron microscopy structure of the human mitoribosome [17], highlighted that the defect was predominantly restricted to the proteins found within the LSU (Figure 4D)

We then examined the 87 proteins that were significantly more abundant in the patient’s fibroblasts. The top Reactome pathways of a greater abundance in the patient fibroblasts (Supplemental Table S5) were cholesterol biosynthesis (via lathosterol and desmosterol, >100-fold enrichment, FDR 4.84 × 10^−2^), semaphorin interactions (34.79-fold enrichment, FDR 2.24 × 10^−2^), and the activation of matrix metalloproteases (33.5-fold enrichment, FDR 1.68 × 10^−4^).

### 2.5. Impaired Growth in Galactose Stress

**Figure 5:**
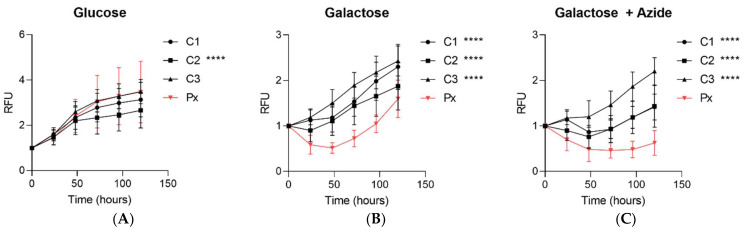
**Galactose stress in patient fibroblasts**. The growth rate in medium devoid of glucose but containing 5 mM galactose and 50 µM sodium azide was normalized to the cell density at T = 0, to account for the minor variation in the seeding density at T = 0. The normalized growth rate of the patient fibroblasts in (**A**) glucose media, (**B**) galactose media, and (**C**) galactose + azide media was significantly lower than the controls. Two-way ANOVA with Dunnett’s multiple comparisons test; pooled data from *n* = 3 independent experiments with 6 measurements/ cell line, **** *p* ≤ 0.0001.

The fibroblasts from the patient and controls were cultured in basal glucose media, galactose media (glucose-free media containing galactose and dialysed serum), or galactose media with a low concentration of the complex-IV inhibitor, sodium azide. When the patient fibroblasts were cultured under basal glucose media, there was no difference in the growth rate for two controls, and one control line grew modestly slower (Figure 5A). However, when the patient fibroblasts were cultured in galactose media, there was a significant decrease in the growth rate of the fibroblasts from the patient compared to all control fibroblasts under galactose conditions (Figure 5B). This impairment in the cell growth was further exacerbated in the galactose media supplemented with a low dose of azide (Figure 5C).

### 2.6. Increased Mitochondrial Superoxide Levels in the PYROXD2 Patient

**Figure 6:**
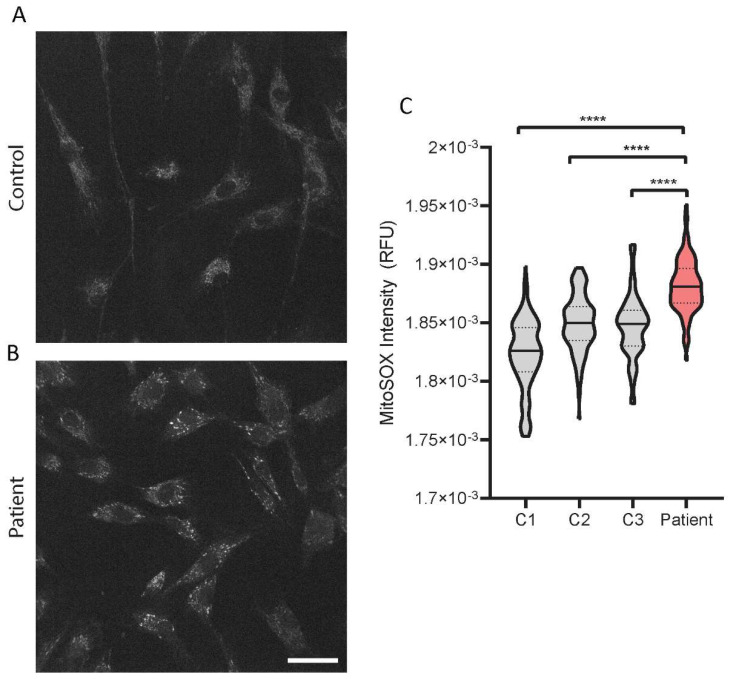
**Mitochondrial superoxide levels in patient fibroblasts**. Patient and control fibroblasts were incubated with the mitochondrial superoxide probe, MitoSOX, prior to imaging with a spinning disc confocal microscope. Representative greyscale images of MitoSOX fluorescent staining for (**A**) one control and (**B**) patient show an increase in MitoSOX intensity in the patient fibroblasts compared to controls. (**C**) Quantification of the relative fluorescent intensity (RFU) in the patient fibroblasts showed a significant increase in the fluorescent intensity in the patient fibroblasts compared to the three paediatric control fibroblasts. All images were taken during the same session with the same microscope parameters. Scale bar represents 50 µm. All images shown have the same intensity settings. Representative data from one experiment from 40–150 cells/sample for three control fibroblast lines and one patient fibroblast line. Repeat experiments show similar results. One-way ANOVA with Sidak’s multiple comparisons test; **** *p* ≤ 0.0001.

We then investigated the levels of mitochondrial superoxide in the patient fibroblasts with a fluorescent probe, MitoSOX, and confocal microscopy. The representative images show the probe accumulating in the mitochondria of the control fibroblasts, with minimal background fluorescence intensity (Figure 6A), and an increased intensity in the patient fibroblasts (Figure 6B). There were significantly increased levels of mitochondrial superoxide levels in *PYROXD2* patient cells compared to control cell lines (Figure 6C).

## 3. Discussion

In this report we demonstrated the critical importance of PYROXD2 in humans. We reported the biallelic variants in *PYROXD2,* discovered by GS analysis in an individual with severe metabolic failure and suspected mitochondrial disease. WGS revealed a missense variant in a highly conserved region, and a frameshift variant altering the last 79 amino acids of PYROXD2. PYROXD2 is part of the pyridine nucleotide-disulfide oxidoreductase domain-containing protein family that regulates redox status and cellular energy. Pyridine nucleotide disulfide reductases catalyse the pyridine nucleotide (NAD/NADH)-dependent reduction of cysteine residues in their substrates and regulate NAD^+^/NADH balance, which are critical mitochondrial metabolites. PYROXD2 is a mitochondrial protein, and emerging evidence shows it has a critical role in mitochondrial function [5].

The clinical presentation of the patient was thought to resemble Leigh syndrome (LS). LS is a neurometabolic disorder affecting the central nervous system, which progresses rapidly and often causes death. The onset of LS is often in infancy, but the disease can occur at any age [3,18]. The most common symptoms of LS are elevated lactate levels in the blood and cerebrospinal fluid, developmental delay, hypotonia, respiratory dysfunction, epileptic seizures, poor feeding, and weakness [18]. The *PYROXD2* patient reported in this study, experienced profound metabolic acidosis, evidence of prior seizures (unusual eye and tongue movements), poor weight gain, hypotonia, and microcephaly. LS is characterised by focal, bilaterally symmetrical, and subacute necrotic lesions in the thalamus, brainstem, and posterior columns of the spinal cord [18]. Cerebral MR imaging in LS patients typically shows symmetric T2-weighted hyperintensities in the striatum, basal ganglia, cerebellum, spinal cord, and brainstem [3,18]. Our patient had bilateral symmetric areas of increased T2-weighted signal evident within the posterior aspect of the midbrain, involving the region of medial longitudinal fasciculus, extending superiorly to the dorsal raphe nuclei, periaqueductal grey, and the central trigeminal tract (CTT).

The appearance of the symmetric, bilateral brainstem changes is suggestive of a toxic or metabolic condition. The restricted diffusion pattern on the diffusion-weighted imaging is particularly suggestive of cytotoxic oedema typically observed in ischaemia/defective oxidative metabolism or direct cytotoxic injury. The features do not conform to any patterns described for a particular metabolic condition, though the involvement of some of these structures are described in specific and non-specific mitochondrial disorders [19]. For example, the involvement of the dorsal midbrain has been described in complex V deficiency, ventral medulla in complex I deficiencies [19], and substantia nigra in Kearns–Sayre syndrome [20]. CTT abnormality has been described in mitochondrial disorders, such as in complex IV (cytochrome *c* oxidase) and complex I deficiencies [19,21], though such changes are non-specific, and is also described in non-mitochondrial metabolic disorders, such as glutaric aciduria Type 1 [21] and non-ketotic hyperglycinaemia [22], childhood epilepsy [21], and in normal children [23]. However, the overall pattern, including the demonstrated progression of change, is most suggestive of a metabolic, and specifically, mitochondrial disorder.

To date, there are no known pathogenic variants reported in *PYROXD2*. However, PYROXD2 and AMP deaminase 3 (AMPD3) were recently shown to be distinctly upregulated in the skeletal muscle of patients with chronic heart failure, which also presented a distinct signature of mitochondrial myopathy and impaired mitochondrial respiration in the same tissue [24]. Several recent genome wide association studies (GWAS) examined the regulation of human serum metabolites in European populations. Positive associations were identified between common single nucleotide polymorphisms in *PYROXD2* and the regulation of the serum metabolome, particularly the effect of *PYROXD2* polymorphisms on trimethylamine (TMA) metabolism [25,26,27,28], which may present an insight into disease-related metabolic deregulation. Trimethylaminuria (TMAU) is a genetic disorder in which the affected individuals cannot convert trimethylamine (TMA) to its oxidized form (TMAO), and variants in the Flavin Containing Dimethylaniline Monoxygenase 3 (*FMO3*) gene are known to be causative. A study of genetically undiagnosed TMAU patients showed an indirect implication of *PYROXD2* in TMA; a common variant identified in GWAS [26] in *PYROXD2* was also identified in many subjects with TMAU [29]. It would be interesting to determine if the association between genetic variation in *PYROXD2* and TMA metabolism extended to the pathogenic variants causing human disease.

Mitochondrial dysfunction is known to be associated with cancer, as the organelle plays major roles in ROS production, resistance to apoptosis, energy production, and cellular proliferation [5]. Therefore, PYROXD2′s potential role in regulating complex IV [5], and hence mitochondrial energy production, could suggest that it possesses a tumour suppressor function. Further evidence includes reduced PYROXD2 protein levels in certain cancer cell lines, such as hepatocellular carcinoma and renal cell carcinoma [30,31,32]. Conversely, the overexpression of *PYROXD2* rescues cancer phenotypes in the cell models described above, primarily by reducing proliferation, increasing apoptosis, and antagonising the effects of the hepatitis B virus X (HBx) protein [30]. These studies have also indicated that PYROXD2 overexpression leads to the increased protein levels of the well-known tumour suppressors, TP53 and p21, and decreases in Cyclin D1 and pRb, leading to reduced cell growth and G1/S cell cycle arrest [31]. PYROXD2 has also been shown to interact with the HBx protein, which is responsible for the oncogenic activity of hepatitis B, and this activity may involve down regulating *PYROXD2* [30,33]. We are not aware of any potentially pathogenic *PYROXD2* variants being reported in human cancer, but it would be of interest to study this.

*PYROXD2* knockout studies highlight the association between PYROXD2 and mitochondrial function, where the reduced mitochondrial membrane potential, decreased ATP levels, cellular proliferation, and mtDNA copy number were observed. *PYROXD2* knockout cell lines also had reduced intracellular ROS, but increased mitochondrial ROS production, indicating an impaired mitochondrial function [5]. To investigate the biological implications of pathogenic *PYROXD2* variants on the mitochondrial function, we used a fluorescent probe, MitoSOX, that is specifically and rapidly targeted to detect mitochondria in live cells. Upon entering the mitochondria, the MitoSOX probe is oxidised by superoxide, but not by other reactive oxygen species or reactive nitrogen species generating systems and produces red fluorescence proportional to the level of superoxide. MitoSOX can be used in conjunction with fluorescence microscopy or flow cytometry, to quantify the mitochondrial superoxide levels in a cell [34]. Higher levels of mitochondrial ROS can indicate cellular stress, and elevated superoxide is often seen in mitochondrial disease. Complex I is the major site of superoxide production in mitochondria, primarily producing superoxide, not hydrogen peroxide, and is a significant contributor to oxidative stress [35], which is interesting since we saw a marked reduction in complex I levels in our proteomics data. Indeed, when we assessed the mitochondrial superoxide levels in the *PYROXD2* patient cells, we found a marked increase in mitochondrial superoxide compared to control fibroblasts, which supports the previous findings that PYROXD2 knockout promotes mitochondrial superoxide production [5]. Elevated superoxide is detrimental to cellular function, and can damage cellular and mitochondrial proteins, cause protein misfolding, mitochondrial energetic failure, and mtDNA damage.

Mitochondrial dysfunction can also be revealed by culturing cells under galactose growth conditions [36], with or without the complex IV inhibitor sodium azide to further enhance the stress [37]. This limits ATP production by glycolysis, forcing the cells to rely on mitochondrial OXPHOS. There was a significant decrease in the growth rate of the fibroblasts from the *PYROXD2* patients, compared to control fibroblasts under galactose conditions. This was further exacerbated by the addition of azide, supporting the idea that mitochondrial function in fibroblasts from PYROXD2 patients is compromised.

PYROXD2 has been found to interact with *COX5B,* a subunit of complex IV, and subsequent *PYROXD2* knockout studies showed a decreased complex IV activity [5]. As complex IV is the terminal complex in the respiratory chain, it is a major site of OXPHOS regulation, and complex IV dysfunction is known to be associated with alterations to cellular proliferation and mitochondrial biogenesis [5]. As part of the clinical diagnosis of this individual, respiratory chain enzymology was undertaken from a muscle biopsy. Respiratory chain enzymes in skeletal muscle homogenates were elevated when expressed relative to protein, and in the normal range when expressed relative to citrate synthase and relative to complex II. Additionally, our proteomics data did not support significant changes to complex IV levels; however, complex I subunit levels were decreased. The enzymology results are not suggestive of a respiratory chain enzyme defect; however, they are unusual in muscles in an infant and suggest the possibility of increased mitochondrial numbers or the increased activity of respiratory chain enzymes. Mitochondrial proliferation is a phenomenon observed in several mitochondrial diseases and is thought to be a compensatory mechanism for reduced mitochondrial efficiency or function caused by variants in key mitochondrial genes. The muscle EM studies did not support a diagnosis of mitochondrial disease in the patient. Given the potential variability in mtDNA levels in control fibroblasts, we did not analyse the mtDNA levels in patient fibroblasts.

The proteomics data interestingly revealed decreased mitoribosomal subunit levels. The mitoribosome provides the protein translation machinery in the mitochondria, and mitoribosomes drive the synthesis of 13 mtDNA encoded proteins of the OXPHOS enzyme complexes. The proteomics data also showed decreased complex I subunit levels, which may be secondary to the mitoribosomal defect. There are numerous examples of mitoribosome defects underlying primary mitochondrial disease [38], and the failure of the mitoribosome leads to respiratory chain failure. Interestingly, the pathogenic variants in several mitoribosomal subunits are reported to cause Leigh syndrome, including variants in *MRPS34* [39] and *PTCD3* [40], which present similarly to the subject presented herein. The primary cause of the changes to the mitoribosomal levels in patient fibroblasts remains to be demonstrated.

**Figure 7:**
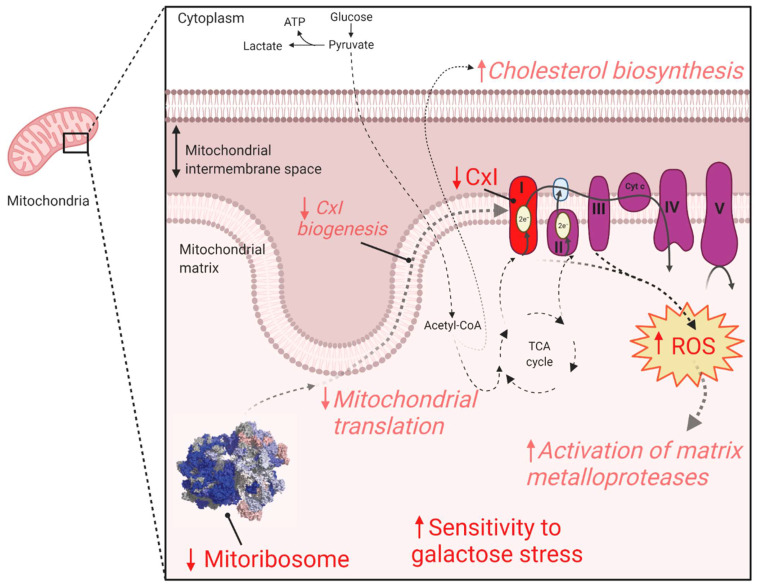
**Schematic of mitochondrial dysfunction in patient fibroblasts**. Quantitative proteomics (see Figure 4) demonstrated decreased levels of several protein subunits of both the large and small subunits of the mitoribosome and complex I (CxI), shown in red. The GO terms enriched in proteins at lower levels in the patient fibroblasts (see Supplemental Table S4) included complex I biogenesis and mitochondrial translation. The GO terms enriched in proteins at higher levels in patient fibroblasts (see Supplemental Table S5) included the activation of matrix metalloproteases and cholesterol biosynthesis. All GO terms are in red italics. Patient fibroblasts were more sensitive to galactose stress and had elevated mitochondrial ROS. Figure created with BioRender.com (accessed on 12 January 2022).

This work provides evidence for the association of biallelic *PYROXD2* variants in causing mitochondrial dysfunction (Figure 7). Our results suggest that the identified variants did not cause overt respiratory chain enzyme activity deficits. However, proteomics analysis revealed significantly reduced complex I and mitoribosomal protein levels, and we demonstrated significantly elevated mitochondrial superoxide levels, together indicative of mitochondrial dysfunction. These mitochondrial defects can explain the increased sensitivity to galactose, a known mitochondrial stressor. Given the above evidence, we suggest that the identified *PYROXD2* variants contribute to mitochondrial disease and are the likely cause of the lethal metabolic disorder in the individual reported here. The identification of additional individuals with biallelic *PYROXD2* variants and similar phenotypes is required to definitively confirm this proposed relationship.

## 4. Materials and Methods

### 4.1. Ethics

All procedures followed were in accordance with the ethical standards and approved by the Human Research Ethics Committee of the Royal Children’s Hospital (HREC/67401/RCHM-2020 and HREC/16/MH251), and in were accordance with the Helsinki Declaration of 1975, as revised in 2000. Written informed consent was obtained from the parents individually and on behalf of their child.

### 4.2. MRI

MRI was performed on a Siemens 3 Tesla system (Magnetom Prisma) and included 2D T2-weighted sequences in standard 3 orthogonal planes (TR = 6940 ms; TE = 170 ms; FA = 120 degrees; in-plane voxel resolution 0.63 mm; slice thickness 2.88 mm); diffusion-weighted imaging (the readout-segment echo-planar imaging (RESOLVE) sequence, 3 b = 1000 s/mm^2^ volumes obtained from 3 orthogonal diffusion-weighted gradient directions, and a single b = 0 s/mm^2^ volume; TR = 2780 ms; TE = 66 ms; in-plane voxel resolution 0.44 mm; slice thickness 3.5 mm); and single-voxel MR spectroscopy (at TE = 144 ms and 30 ms).

### 4.3. Biological Samples

Genomic DNA was isolated from whole blood cells of the reported cases and their parents using the QIAamp DNA mini kit (Qiagen, Hilden, Germany), following the manufacturer’s instructions and used for GS. RNA from blood was used for RNA sequencing. RNA from skin fibroblasts cultured from Individual 1 was extracted using a commercially available kit (Qiagen RNeasy kit, Qiagen, Hilden, Germany) for RT-PCR and qPCR. Sanger sequencing was completed in a genomic DNA sample from the individual’s fibroblasts to confirm the presence of both *PYROXD2* variants in the established fibroblast line. A muscle biopsy was preserved in glutaraldehyde and processed in resin for electron microscopy.

### 4.4. Genome Sequencing

GS was performed by the Victorian Clinical Genetics Services using clinically accredited processes for massively parallel sequencing (NexteraTM DNA Flex Library Prep kit, Illumina Sequencers, San Diego, CA, USA) with a mean target coverage of 30× and a minimum of 90% of bases sequenced to at least 10×. Data was processed, including the read alignment to the reference genome (GRCh38) and variant calling, using Cpipe [41], or via functionally equivalent analysis with the Illumina Dragen System. Automated sex determination, relatedness, and contamination checks were performed.

### 4.5. RNA Sequencing (RNAseq)

RNA for individual 1 was extracted from whole blood using the Omega Bio-Tek E.Z.N.A DNA/RNA Isolation kit, according to the manufacturer’s instructions with an elution volume of 40 uL. RNA quantification was performed using the Invitrogen Qubit RNA kit and RNA integrity was checked for suitability in RNAseq using the Agilent TapeStation RNA High Sensitivity buffers and tapes (TapeStation 2200 and 4200, Agilent Technologies Australia, VIC, Australia). For this family, 58–77.9 ng of total RNA (10 uL of RNA of 5.8–7.79 ng/uL) were used as input for the Illumina TruSeq Stranded Total RNA with Ribo-Zero Globin depletion library prep.

Library QCs were performed using the Agilent TapeStation D1000 High Sensitivity buffers and tapes or the QIAGEN QIAexcel system. Libraries were sequenced on the Illumina NovaSeq 6000 using 2 × 150 bp reads and with a targeted sequencing depth of 80 M fragments/160 M paired-end reads per library. RNASeq data was trimmed using Trimmomatic 0.39 and aligned using STAR 2.7.3a to GRCh38 with transcript annotations from Gencode v37. The alignments were visualised using the Integrated Genomics Viewer (IGV) 2.4.13.

### 4.6. Variant Analysis

The initial GS variant analysis and interpretation was performed by the Victorian Clinical Genetics Services using clinically accredited processes in Agilent Alissa Interpret, as previously described [6]. Copy number variants (CNVs) were screened for using an internal CNV detection tool, CXGo [42]. Mitochondrial DNA analysis from the GS data was performed in-house with mitochondrial data analysis and annotation tools.

*PYROXD2* was submitted to Matchmaker Exchange [43], an international database to match subjects of similar phenotype and potential pathogenic variants, but no matches with compatible phenotypes or variant spectrum were identified as of 1 October 2021.

The 3D protein structure of PYROXD2 was predicted by AlphaFold (AF-Q8N2H3-F1) and was downloaded from Uniprot (23 September 2021). The 3D model was uploaded to the viewer NCBI icn3d (https://www.ncbi.nlm.nih.gov/Structure/icn3d/full.html, accessed on 23 September 2021) and the missense variant and frameshift regions were highlighted. The linear protein ribbon structure overlayed on the amino acid sequence was also captured from icn3d.

### 4.7. Cell Culture

The primary cultures of fibroblasts from the patient and unrelated control paediatric fibroblasts were established from skin biopsies, as previously described [44]. The fibroblasts were cultured in basal medium (high-glucose DMEM (Gibco, Waltham, MA, USA) with 10% foetal bovine serum (Gibco), 100 units/mL penicillin, and 100 µg/mL streptomycin) at 37 °C with 5% CO_2_. All the fibroblast control cell lines were established in-house and were established from paediatric individuals without any suspected genetic disorders.

### 4.8. RT-PCR and qPCR Analysis

Total RNA was isolated using a commercially available kit (Qiagen RNeasy kit). cDNA was synthesized (GoScript Reverse Transcription Mastermix with random hexamers, Promega, Madison, WI, USA). RT-PCR reactions were performed with custom primers and Platinum Taq Mastermix (Invitrogen, Waltham, Massachusetts, USA). Quantitative reverse-transcription PCR (qPCR) reactions were performed using custom primers and AccuPower 2X Greenstar qPCR Master Mix (Bioneer Pacific, Kew, VIC, Australia) and a Roche Real Time PCR machine.

### 4.9. Immunoblotting

For denaturing gels, the fibroblasts were extracted in RIPA buffer (10 mM Tris-Cl (pH 8.0), 1 mM EDTA, 0.5 mM EGTA, 1% Triton X-100, 0.1% sodium deoxycholate, 0.1% SDS, 140 mM NaCl, 1 mM PMSF, and protease inhibitor cocktail (Roche, Basel, Switzerland)) with gentle sonication, then incubated on ice for 30 min prior to centrifugation at 20,000× *g* for 20 min at 4 °C. The protein concentration was determined using the Pierce BCA Protein Assay Kit. SDS-PAGE was performed on 10–30 µg of cell lysate per sample using standard techniques on Tris-glycine-SDS polyacrylamide gels (Biorad, Hercules, CA, USA). The primary antibodies used were against the mitochondrial respiratory chain subunits (mitochondrial OXPHOS antibody cocktail containing cytochrome c oxidase subunit 2 (COX2), cytochrome b-c1 complex subunit 2 (UQCRC2), succinate dehydrogenase (ubiquinone) flavoprotein subunit B (SDHB), NADH dehydrogenase (ubiquinone) 1 beta subcomplex subunit 8 (NDUFB8), and ATP synthase subunit alpha (ATP5A); Cat. #ab110411, Abcam, 1:500), PYROXD2 (Cat. # ab228998, Abcam, 1:1500), and GAPDH detection (antibody Cat. #G9545) was used to normalize for total cellular protein. The primary antibodies were detected with appropriate anti-mouse or anti-rabbit horseradish peroxidase-conjugated antibodies (GE Healthcare, Livingston, NJ, U.S.A.), using enhanced chemiluminescence reagents (Biorad Clarity Western ECL substrate) and Amersham Hyperfilm. The protein band intensities were quantified using ImageJ software [45], and band intensity determined in the linear range was normalized to band intensity of GAPDH.

### 4.10. Spectrophotometric Respiratory Chain Enzyme Assays

Skeletal muscle post 600 g supernatants or enriched fibroblast mitochondria were used to measure specific activity of complexes I, II, II + III, III, IV, and citrate synthase, as previously described [10]. The data were normalized to protein concentration and to the activity of complex II or citrate synthase. The duplicate measurements were taken for each sample, and each cell line or muscle biopsy was assayed in duplicate.

### 4.11. Dipstick Respiratory Chain Enzyme Assays

Protein from whole cell lysates was extracted in a cell extraction buffer, following the manufacturer’s instructions (catalogue numbers ab109720 and ab109876; MitoSciences, Eugene, OR, USA.) plus protease inhibitors (Thermo Scientific, Waltham, Massachusetts, United States) and protein concentration determined with BCA assay (Pierce). Complex I and complex IV dipstick activity assays were performed using 15 μg of protein for complex I, and 20 μg of protein for complex IV. The dipsticks were then scanned using a high-resolution flatbed scanner (Canon CS9000F MKii, Canon, Ota City, Tokyo, Japan) for signal intensity quantitation. The data were normalized to protein concentration. Duplicate measurements were taken for each sample, and each cell line was assayed in at least triplicate.

### 4.12. Quantitative Proteomics

Fibroblasts cell pellets were solubilised in 5% SDS (*v*/*v*), 50 mM triethylammonium bicarbonate (TEAB) and quantified using a PierceTM BCA protein assay kit (Thermo Fischer Scientific). A total of 20 µg of protein from the *PYROXD2* patient (in triplicate) and 5 independent normal controls (in singlicate) were prepared using S-trapTM micro spin column protocol, as per manufacturer’s instructions. The proteins were digested at 1:10 trypsin to protein ratio at 37 °C overnight. Peptides were eluted with three elution steps as the protocol. The samples were dried down using a CentriVap Benchtop Vacuum Concentrator (Labconco, Kansas City, MO, USA) and reconstituted in 2% acetonitrile (ACN) 0.1% trifluoroacetic acid (TFA) for LC-MS/MS.

The LC system was equipped with an Acclaim Pepmap nano-trap column (Dinoex-C18, 100 Å, 75 µm × 2 cm) and an Acclaim Pepmap RSLC analytical column (Dinoex-C18, 100 Å, 75 µm × 50 cm). The tryptic peptides were injected into the enrichment column at an isocratic flow of 5 µL/min of 2% *v*/*v* ACN containing 0.1% *v*/*v* formic acid for 5 min, applied before the enrichment column was switched in-line with the analytical column. The eluents were 5% dimethyl sulfoxide (DMSO) in 0.1% *v*/*v* formic acid (solvent A), 5% DMSO in 100% *v*/*v* ACN, and 0.1% v/v formic acid (solvent B). The flow gradient was (i) 0–6 min at 3% B; (ii) 6–7 min 3–4%; (iii) 7–82 min, 4–25% B; (iv) 82–86 min, 25–40% B; (v) 86–87 min, 40–80% B; (vi) 87–90 min, 80–80% B; (vii) 90–95 min, 80–3%; and equilibrated at 3% B for 10 min before the next sample injection. For the DIA experiments, full MS resolutions were set to 120,000 at m/z and scanning from 350–1400 m/z in the profile mode. The full MS AGC target was 250% with an IT of 45 ms. The AGC target value for fragment spectra was set at 1000%. A total of 50 windows of 13.7 Da were used with an overlap of 1 Da. The resolution was set to 15,000 and maximum IT to 22 ms. The normalized collision energy was set at 30%. All the data were acquired in centroid mode using positive polarity.

DIA files were processed with Spectronaut (v.115.2.210819.50606, Rubin) against a DDA library containing 124,877 precursors from deep fractionated fibroblasts samples. Default BSG Factory search parameters were used with a few modifications. “Major Group Top N” and “Minor Group Top N” options were unselected to allow all peptides to be considered for quantitation and “Data filtering option” was set to “Q-value”. The protein search was performed using UniProt reviewed human canonical and isoform (42,360 entries). Data processing and statistical analysis were performed in Perseus (v.1.6.14.0) [46]. MS2 quantities were log2-transformed and samples were labelled into their respective groups (PYROXD2 or Controls). The groups were filtered to have least two valid values for the two-sample *t*-test which used a *p*-value for truncation (threshold *p*-value = 0.05). The volcano plots were generated using the scatter-plot function in Perseus and the significance lines were set to *p*-value = 0.05 (-log10 = 1.301) and fold-change ± 2 (log2 ± 1). The MitoCarta 3.0 database [47] was used to annotate the entries using the UniProt IDs column. Relative complex abundance (RCA) plots were generated in R (RStudio), calculating the difference between the PYROXD2 and the controls for each subunit of the complex. The mean and standard deviation were calculated from the MS2 quantities, along with the confidence interval based on the *t*-statistic for each complex (which was calculated from the difference between the control and patient samples). A paired *t*-test calculated significance between the control and patient for each complex. The topographical mapping of differential protein abundances from the *t*-test were mapped to the mitoribosome structure (PDB id: 3J9M), as previously described [48].

The gene ontology (GO) terms for the significantly altered proteins were extracted, and the overrepresented functional categories for the differentially abundant proteins were determined by the Gene Ontology Resource (http://geneontology.org/, accessed on 15 September 2021) Enrichment Analysis Tool [14,15,16]. The reference list was *Homo sapiens* (all genes in the online database; data accessed 2019). All the proteins that were subjected to SpI analysis served as the background list, and GO terms with at least five proteins were used for the statistical calculations. A *p*-value for each term was calculated via the one-sided Fisher’s exact test, and the FDR was estimated by the software.

### 4.13. Metabolic Stress Assays

Fibroblast cells were seeded in basal media at 1 × 10^4^ cells/well into a 96-well dish and allowed to attach overnight. The following day, media was replaced with either 100 µL of DMEM (high-glucose) supplemented with 10% foetal bovine serum (FBS); 2.05 mM L-glutamine; 100 units/mL penicillin; and 100 µg/mL streptomycin (basal medium) or 100 µL of DMEM (glucose-free) supplemented with 5 mM galactose, 10% dialyzed FBS, 2.05 mM L-glutamine, 100 units/mL penicillin, and 100 µg/mL streptomycin (metabolic stress medium), with or without 50 µM sodium azide. Cell growth was monitored daily using the cell-permeable fluorescent dye, resazurin. At each time point, resazurin (20 µL/well of 0.15 mg/mL solution) was added to each well and incubated at 37 °C with 5% CO_2_ for 4 h prior to measuring the relative fluorescent units (RFU; Ex = 530–570 nm, Em = 590–620 nm). The media-only values were subtracted from each reading on each day. The RFU data at each time point was expressed relative to the untreated cells on day 0 [49].

### 4.14. Mitochondrial Superoxide Staining

Fibroblast cells were plated onto glass-bottomed confocal dishes and allowed to attach overnight at 37 °C and 5% CO_2_. The following day, a fresh tube of MitoSOX Red (M36008, Thermofisher) was resuspended in DMSO, then added to Hankʼs balanced salt solution with calcium and magnesium (HANKS; Gibco (14025-092)) to 5 µM and warmed to 37 °C. The media was removed from the culture dish and replaced with the MitoSOX staining solution, and incubated in the dark at 37 °C for 10 min. The cells were washed 3x with pre-warmed HANKS, then imaged in HANKS. Fluorescent intensity in the cells was captured using a 40× oil-immersion objective, using an Andor Dragonfly spinning disc confocal microscope with a sCMOS camera connected to Imaris software (Oxford Instruments, Abingdon, UK). The environment was controlled with a heated stage set at 37 °C and a CO_2_ chamber set to 5%. All microscope image capture settings remained consistent throughout each imaging session. For each cell line, two dishes were imaged during each imaging session, with a minimum of 6 random areas captured per dish, totalling between 40–150 cells per sample per session.

### 4.15. Image Analysis

MitoSOX-stained cell images were processed with a pipeline function generated in CellProfiler (Version 4.0.7, Broad Institute, Cambridge, MA, USA) [50]. Masks were generated for the cell, to measure the intensity, after the background correction of each image. The mean intensity was measured for each masked region.

### 4.16. Statistical Analysis

Statistical analyses were carried out using either a two-tailed Student’s *t*-test or one-way ANOVA corrected for multiple comparisons, as appropriate (GraphPad Prism^®^ Software, San Diego, CA, USA). The error bars represent the standard deviation of the mean (±SD). A *p*-value < 0.05 was considered statistically significant.

### 4.17. Data Availability

The de-identified raw data that support the findings of this study are available from the corresponding author, upon request. The mass spectrometry proteomics data will be deposited in the ProteomeXchange Consortium via the PRIDE PRoteomics IDEntifications (PRIDE) Archive database [51] partner repository upon publication.

## Data Availability

The de-identified raw data that support the findings of this study are available from the corresponding author, upon request. The mass spectrometry proteomics data will be deposited to the ProteomeXchange Consortium via the PRIDE [51] partner repository upon publication.

## Supplementary Materials

The following are available online at https://www.mdpi.com/article/10.3390/ijms23020986/s1. Reference [52] is cited in the supplementary materials.
