# Supplementary material for: Biallelic Variants in PYROXD2 Cause a Severe Infantile Metabolic Disorder Affecting Mitochondrial Function
Source: Int J Mol Sci. 2022 Jan 17;23(2):986. doi: 10.3390/ijms23020986 (PMC8777681; doi:10.3390/ijms23020986)
Supplement: Supplementary file 1 [file ijms-23-00986-s001.zip › PYROXD2 manuscript_IJMSformat_SUBMITTED_Supplemental.pdf]

International Journal of
Molecular Sciences

MDPI

Appendix A: Supplemental material

Article: Biallelic variants in PYROXD2 cause a severe infantile metabolic disorder affecting mitochondrial function

Supplemental Table 1. Respiratory chain enzyme activities in skeletal muscle biopsy of *PYROXD2* patient.

	**Activity**		**Ref. Range**	**% Activity**	**% CS Ratio**	**% CII Ratio**
**Complex I** (NADH-coenzyme Q_1 oxidoreductase)	134	nmol/min/mg	(19 - 72)	327	121	132
**Complex II** (Succinate-coenzyme Q_1 oxidoreductase)	112	nmol/min/mg	(26 - 63)	248	91	
**Complex II+III** (Succinate cytochrome c reductase)	72	nmol/min/mg	(30 - 76)	157	59	64
**Complex III** (Decylbenzylquinol-cytochrome *c* oxidoreductase)	80.3	/min/mg	(13 - 51)	277	97	108
**Complex IV** (Cytochrome *c* oxidase)	10.84	/min/mg	(3.3 - 9.1)	164	61	67
**Citrate Synthase**	349	nmol/min/mg	(85 - 179)	271		

Note: All measurements are performed in duplicate and were only accepted when each of the duplicate values was within a 10% range of their average. Enzyme activities are shown as absolute values and as % residual activity relative to protein (% Activity), Citrate Synthase (% CS Ratio), and Complex II (% CII Ratio). % values ≤ 30 are shown in bold and represent major or minor criteria in the Bernier Diagnostic Scheme [1].

Supplemental Table 2. Respiratory chain enzyme activities in skin fibroblasts of *PYROXD2* patient.

	Activity		Ref. Range	% Activity	% CS Ratio	% CII Ratio
**Complex I** (NADH-coenzyme Q[1] oxidoreductase)	176	nmol/min/mg	(93 – 131)	150	85	90
**Complex II** (Succinate-coenzyme Q_1 oxidoreductase)	162	nmol/min/mg	(87 – 120)	164	95	
**Complex II+III** (Succinate cytochrome *c* reductase)	169	nmol/min/mg	(70 - 79)	223	128	133
**Complex III** (Decylbenzylquinol-cytochrome *c* oxidoreductase)	39.2	/min/mg	(21.2 - 26.2)	161	94	97
**Complex IV** (Cytochrome *c* oxidase)	7.53	/min/mg	(5.28 - 5.82)	136	78	81
**Citrate Synthase**	471	nmol/min/mg	(215 - 319)	169		

Note: All measurements are performed in duplicate and were only accepted when each of the duplicate values was within a 10% range of their average. Enzyme activities are shown as absolute values and as % residual activity relative to protein (% Activity), Citrate Synthase (% CS Ratio), and Complex II (% CII Ratio). % values ≤ 30 are shown in bold and represent major or minor criteria in the Bernier Diagnostic Scheme [1].

Supplemental Table 3. Proteomics data on *PYROXD2* patient, including data used to generate Volcano plots and profile plots of log2-transformed intensities for cellular proteins in patient and control fibroblasts.

(SEE EXCEL SPREADSHEET)

Supplemental Table 4. GO terms enriched in proteins of lower level in patient fibroblasts compared to controls.

**Reactome pathways**	**Homo sapiens - REFLIST (20595)**	**Uploaded (272)**	**Uploaded (expected)**	**Uploaded (over /under)**	**Uploaded fold Enrichment)**	**Uploaded (raw P-value)**	**uploaded (FDR)**
Mitochondrial translation elongation (R-HSA-5389840)	88	16	1.16	+	13.77	4.25E-13	9.71E-10
Mitochondrial translation termination (R-HSA-5419276)	88	16	1.16	+	13.77	4.25E-13	4.85E-10
Mitochondrial translation initiation (R-HSA-5368286)	88	16	1.16	+	13.77	4.25E-13	3.24E-10
Mitochondrial translation (R-HSA-5368287)	94	16	1.24	+	12.89	1.04E-12	4.76E-10
Complex I biogenesis (R-HSA-6799198)	55	8	0.73	+	11.01	1.57E-06	5.96E-04
Striated Muscle Contraction (R-HSA-390522)	36	5	0.48	+	10.52	1.85E-04	3.84E-02
Smooth Muscle Contraction (R-HSA-445355)	39	5	0.52	+	9.71	2.60E-04	4.95E-02
Cargo recognition for clathrin-mediated endocytosis (R-HSA-8856825)	104	9	1.37	+	6.55	1.74E-05	4.98E-03
Translation (R-HSA-72766)	293	25	3.87	+	6.46	7.50E-13	4.28E-10
Respiratory electron transport (R-HSA-611105)	100	8	1.32	+	6.06	8.59E-05	2.18E-02
Clathrin-mediated endocytosis (R-HSA-8856828)	144	9	1.9	+	4.73	1.85E-04	4.23E-02
Metabolism of proteins (R-HSA-392499)	1977	51	26.11	+	1.95	4.23E-06	1.38E-03

Supplemental Table 5. GO terms enriched in proteins of higher level in patient fibroblasts compared to controls.

**Reactome pathways**	**Homo sapiens - REFLIST (20595)**	**Uploaded (272)**	**Uploaded (expected)**	**Uploaded (over /under)**	**Uploaded fold Enrichment)**	**Uploaded (raw P-value)**	**uploaded (FDR)**
Cholesterol biosynthesis via lathosterol (R-HSA-6807062)	4	2	0.02	+	> 100	2.96E-04	4.84E-02
Cholesterol biosynthesis via desmosterol (R-HSA-6807047)	4	2	0.02	+	> 100	2.96E-04	4.51E-02
Cholesterol biosynthesis (R-HSA-191273)	24	8	0.11	+	73.82	1.18E-12	2.70E-09
Other semaphorin interactions (R-HSA-416700)	19	3	0.09	+	34.97	1.27E-04	2.24E-02
Activation of Matrix Metalloproteinases (R-HSA-1592389)	33	5	0.15	+	33.55	7.35E-07	1.68E-04
Activation of gene expression by SREBF (SREBP) (R-HSA-2426168)	40	6	0.18	+	33.22	5.68E-08	3.24E-05
Regulation of cholesterol biosynthesis by SREBP (SREBF) (R-HSA-1655829)	53	6	0.24	+	25.07	2.61E-07	9.92E-05
Collagen degradation (R-HSA-1442490)	64	6	0.29	+	20.76	7.29E-07	1.85E-04
Metabolism of steroids (R-HSA-8957322)	148	10	0.67	+	14.96	2.38E-09	2.72E-06
Integrin cell surface interactions (R-HSA-216083)	84	5	0.38	+	13.18	5.08E-05	9.66E-03
Degradation of the extracellular matrix (R-HSA-1474228)	140	8	0.63	+	12.65	3.46E-07	9.87E-05
Extracellular matrix organization (R-HSA-1474244)	299	11	1.35	+	8.15	1.47E-07	6.70E-05
Metabolism of lipids (R-HSA-556833)	733	18	3.31	+	5.44	5.55E-09	4.23E-06
Metabolism (R-HSA-1430728)	2079	27	9.39	+	2.88	3.31E-07	1.08E-04
Unclassified (UNCLASSIFIED)	9941	23	44.89	-	0.51	5.29E-06	1.10E-03

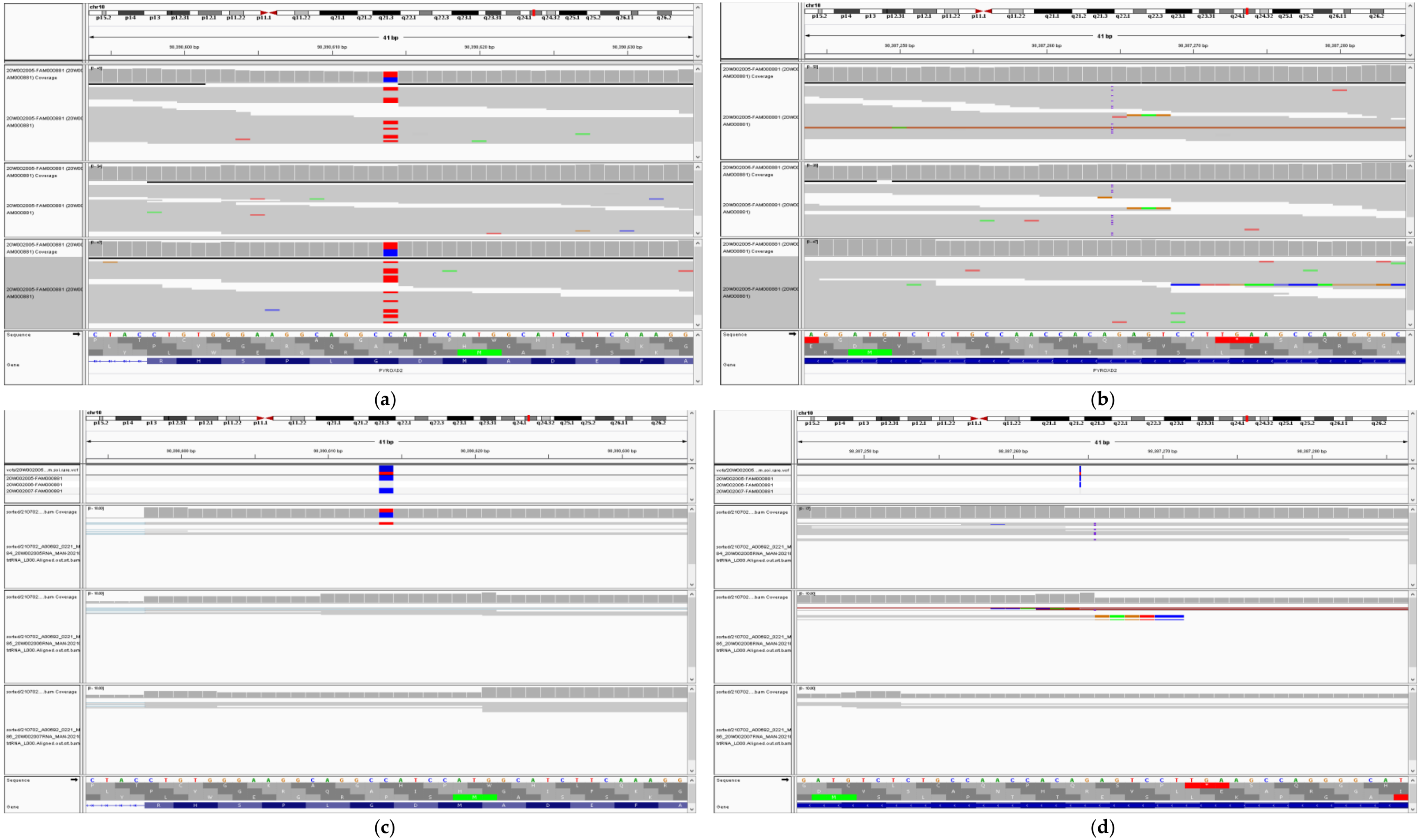

Supplemental Figure 1. GS and RNAseq analysis of *PYROXD2* in patient fibroblasts. (A) The paternally inherited PYROXD2 missense variant (NM_32709.2: c.1276G>A; p.(Gly426Ser); and **(B)** the maternally inherited frameshift variant (NM_32709.2: c.1490dupC, p.(Val498Cysfs*79) from GS data. **(C)** RNAseq analysis confirmed the missense variant was expressed in the proband in approximately 50% of the observed reads; and **(D)** the frameshift variant was observed in the RNASeq data in both the proband and the mother in approximately 50% of the reads.

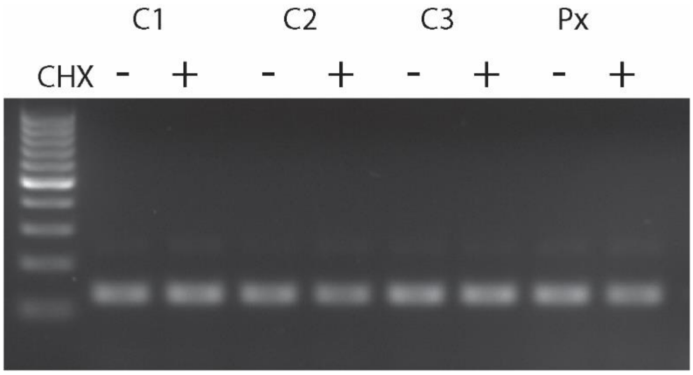

(a)

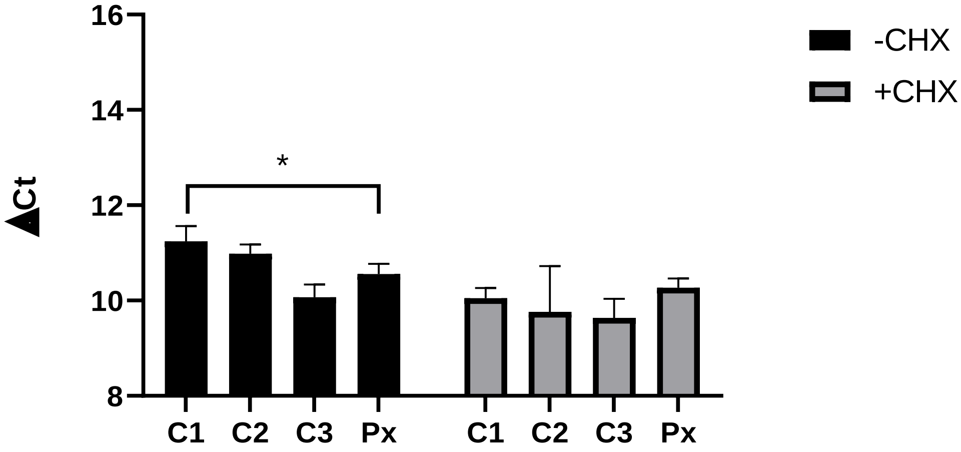

(b)

Supplemental Figure 2. RT-PCR and qPCR of *PYROXD2* in patient fibroblasts. (A) RT-PCR analysis shows similar levels of PYROXD2 transcript in patient and controls, with (+) or without (-) cycloheximide. **(B)** qPCR of PYROXD2 show no difference in PYROXD2 levels between two of the three controls (C1 - C3) and patient (Px) fibroblasts. * $p <= 0.05$.

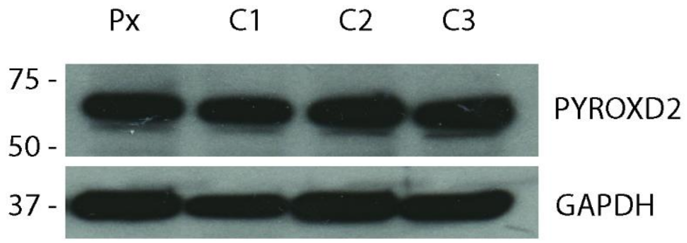

Supplemental Figure 3. Western blot of PYROXD2 in patient fibroblasts. Western blot of PYROXD2 in patient fibroblasts (Px) and three controls (C1 - C3) showing normal levels of PYROXD2 protein.

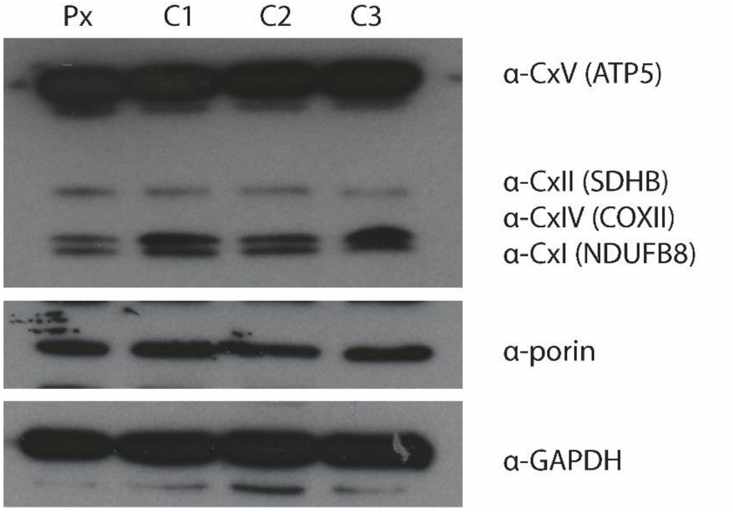

Supplemental Figure 4: Western blot of OXPHOS complex subunits in patient fibroblasts. Western blot of a representative subunit of each OXPHOS complex including; cytochrome c oxidase subunit 2 [COX2], cytochrome b-c1 complex subunit 2 [UQCRC2], succinate dehydrogenase [ubiquinone] flavoprotein subunit B [SDHB], NADH dehydrogenase [ubiquinone] 1 beta subcomplex subunit 8 [NDUFB8] and ATP synthase subunit alpha [ATP5A] in patient fibroblasts (Px) and three controls (C1 - C3) showing normal levels of OXPHOS subunit proteins.

References

1. Bernier, F.P.; Boneh, A.; Dennett, X.; Chow, C.W.; Cleary, M.A.; Thorburn, D.R. Diagnostic criteria for respiratory chain disorders in adults and children. *Neurology* **2002**, *59*, 1406-1411, doi:10.1212/01.wnl.0000033795.17156.00.
