# Supplementary material for: Biallelic Variants in PYROXD2 Cause a Severe Infantile Metabolic Disorder Affecting Mitochondrial Function
Source: Int J Mol Sci. 2022 Jan 17;23(2):986. doi: 10.3390/ijms23020986 (PMC8777681; doi:10.3390/ijms23020986)

International Journal of
Molecular Sciences

Attachment: Full western blots from data in article

Article: Biallelic variants in PYROXD2 cause a severe infantile metabolic disorder affecting mitochondrial function

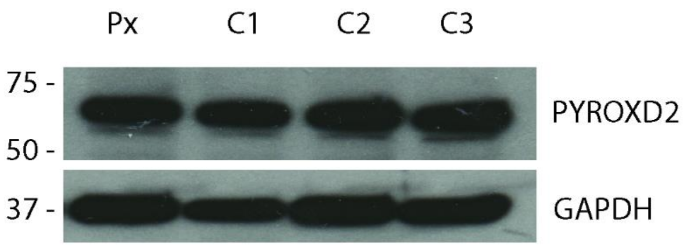

Supplemental Figure 3. Western blot of PYROXD2 in patient fibroblasts. Western blot of PYROXD2 in patient fibroblasts (Px) and three controls (C1 - C3) showing normal levels of PYROXD2 protein.

Full Western blots below

Anti-PYROXD2

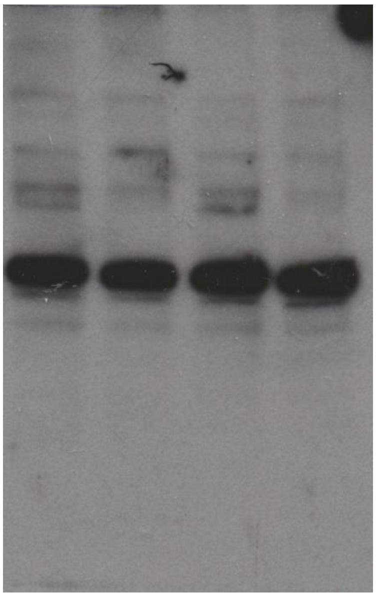

Anti-GAPDH (indicated with arrow)

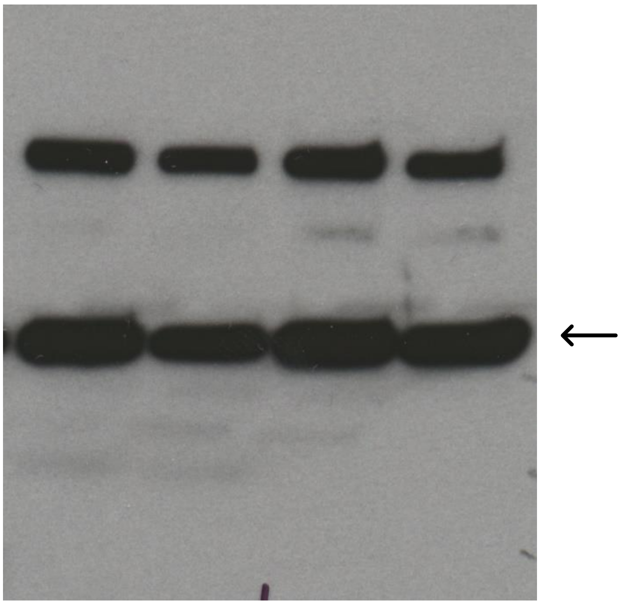

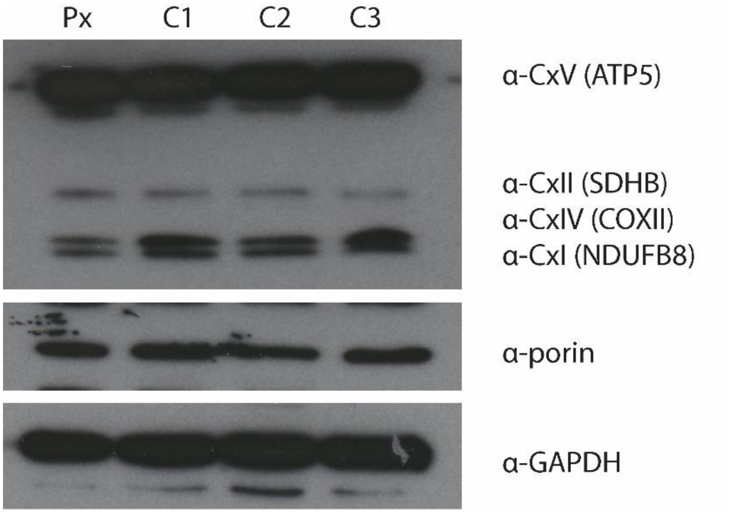

Supplemental Figure 4: Western blot of OXPHOS complex subunits in patient fibroblasts. Western blot of a representative subunit of each OXPHOS complex including; cytochrome c oxidase subunit 2 [COX2], cytochrome b-c1 complex subunit 2 [UQCRC2], succinate dehydrogenase [ubiquinone] flavoprotein subunit B [SDHB], NADH dehydrogenase [ubiquinone] 1 beta subcomplex subunit 8 [NDUFB8] and ATP synthase subunit alpha [ATP5A] in patient fibroblasts (Px) and three controls (C1 - C3) showing normal levels of OXPHOS subunit proteins.

Full Western blots

Anti-OXPHOS (CxV, CxII, CxIV and CxI)

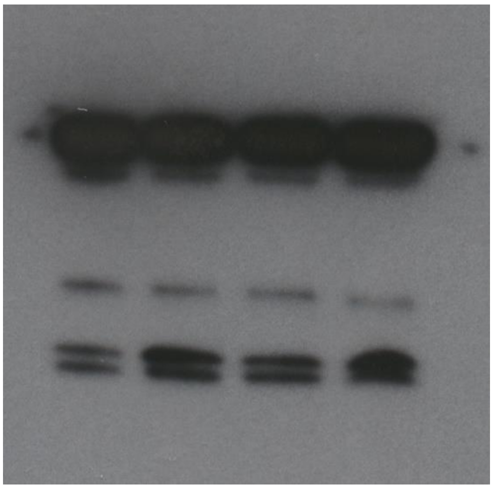

Anti-Porin (after anti-OXPHOS probing). Porin indicated with an arrow

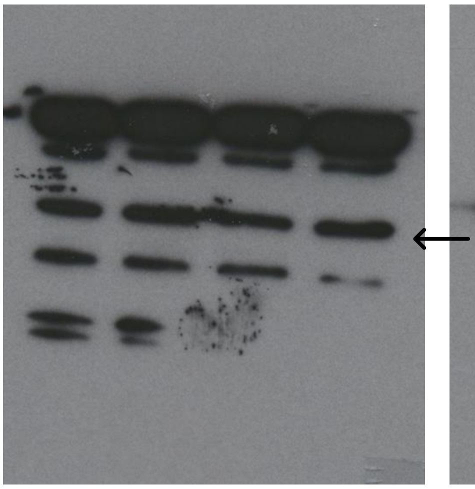

Anti-GAPDH

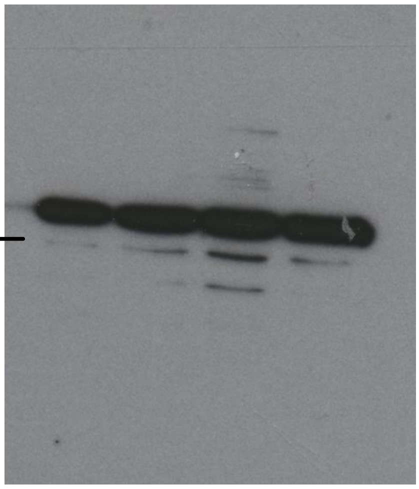

Supplement: Supplementary file 1 [file ijms-23-00986-s001.zip › PYROXD2 manuscript_IJMSformat_SUBMITTED_WesternBlotFull.pdf]
